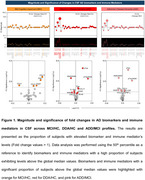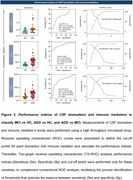# Cerebrospinal biomarkers and immune mediator profiles across clinical stages of Alzheimer’s disease in a sample of Brazilian older adults

**DOI:** 10.1002/alz70861_108835

**Published:** 2025-12-23

**Authors:** Ivonne Carolina Bolaños Burgos, Júlia de Almeida Barreto, Caio Mendes Ribeiro, Pedro Henrique Oliveira Aquino, Gabriela Tomé Oliveira Engelmann, Erika de Oliveira Hansen, Natália Silva Dias, Debora Marques de Miranda, Marco Aurélio Romano‐Silva, Luiz Armando Cunha de Marco, Christiane Costa Pereira, Andréa Teixeira Carvalho, Olindo Assis Martins‐Filho, Bernardo de Mattos Viana, Maria Aparecida Camargos Bicalho

**Affiliations:** ^1^ graduate program in applied sciences to adult health, Belo Horizonte, Minas Gerais Brazil; ^2^ Cog‐Aging Research Group, Belo Horizonte, Minas Gerais Brazil; ^3^ National Institute of Science and Technology Neurotec R (INCT‐MM) Faculdade de Medicina, Universidade Federal de Minas Gerais, Belo Horizonte Brazil; ^4^ Neurotec R National Institute of Science and Technology (INCT‐Neurotec R), Faculty of Medicine, Universidade Federal de Minas Gerais (UFMG), Belo Horizonte, Minas Gerais Brazil; ^5^ Undergraduate Medicine, Faculty of Medicine, Universidade Federal de Minas Gerais (UFMG), Belo Horizonte, Minas Gerais Brazil; ^6^ Undergraduate medicine, Faculty of Medicine, Universidade Federal de Minas Gerais (UFMG), Belo Horizonte, Minas Gerais Brazil; ^7^ Neurotec R National Institute of Science and Technology (INCT‐Neurotec R), Faculty of Medicine, Universidade Federal de Minas Gerais (UFMG), Belo Horizonte, Minas Gerais Brazil; ^8^ Cog‐Aging Research Group, 31, Minas Gerais Brazil; ^9^ Cog‐Aging Research Group, Universidade Federal de Minas Gerais (UFMG), Belo Horizonte, Minas Gerais Brazil; ^10^ Molecular Medicine Postgraduate Program, School of Medicine, Universidade Federal de Minas Gerais (UFMG), Belo Horizonte, Minas Gerais Brazil; ^11^ Geriatrics and Gerontology Center Clinical Hospital of Universidade Federal de Minas Gerais, Belo Horizonte, Minas Gerais Brazil; ^12^ Department of Mental Health, Faculty of Medicine, Universidade Federal de Minas Gerais (UFMG), Belo Horizonte, Minas Gerais Brazil; ^13^ Department of Psychiatry, School of Medicine, Federal University of Minas Gerais, Belo Horizonte, Minas Gerais Brazil; ^14^ Molecular Medicine Postgraduate Program, Faculty of Medicine, Universidade Federal de Minas Gerais (UFMG), Belo Horizonte, Minas Gerais Brazil; ^15^ René Rachou Institute, Oswaldo Cruz Foundation (Fiocruz), Belo Horizonte, Minas Gerias Brazil; ^16^ René Rachou Institute, Oswaldo Cruz Foundation (Fiocruz), Belo Horizonte, Minas Gerais Brazil; ^17^ René Rachou Institute, Oswaldo Cruz Foundation (Fiocruz), Belo Horizonte, Minas Gerias Brazil; ^18^ National Institute of Science and Technology Neurotec R (INCT‐MM), Faculdade de Medicina, Universidade Federal de Minas Gerais, Belo Horizonte Brazil; ^19^ Older Adult’s Psychiatry and Psychology Extension Program (PROEPSI), School of Medicine, Universidade Federal de Minas Gerais (UFMG), Belo Horizonte, Minas Gerais Brazil; ^20^ Department of Clinical Medicine, Faculty of Medicine, Universidade Federal de Minas Gerais (UFMG), Belo Horizonte, Minas Gerais Brazil; ^21^ Sciences Applied to Adult Health Postgraduate Program, School of Medicine, Universidade Federal de Minas Gerais (UFMG), Belo Horizonte, Minas Gerais Brazil

## Abstract

**Background:**

The neuroinflammatory process in Alzheimer’s disease (AD) is characterized by increased reactivity of astrocytes and microglia, along with elevated production and release of immune mediators. Studies have shown that the reactive microglial phenotype in AD contributes to heightened levels of these mediators, which in turn exacerbate amyloid and tau pathology, ultimately leading to a sustained and robust inflammatory response. Brazil’s genetically diverse population offers a unique opportunity to investigate these immune mechanisms, potentially revealing population‐specific inflammatory profiles. This study aims to profile Alzheimer's disease biomarkers and immune mediators across clinical stages of the disease in a cohort of Brazilian older adults.

**Method:**

One hundred and eight older adults were recruited and categorized based on clinical profiles in three different groups: Alzheimer’s Disease Dementia (ADD) (*n* =54), Mild Cognitive Impairment (MCI) (*n* =33), and Healthy Controls (HC) (*n* =21). Cerebrospinal fluid (CSF) Aβ42, *p* ‐Tau, t‐Tau, *p* ‐Tau/ Aβ42 and Aβ42/ Aβ40 and 27 immune mediators were assessed using the Luminex xMAP technique. Fold change analyses were conducted to identify differential expression patterns of biomarkers and immune mediators across clinical stages. To evaluate their discriminatory potential, receiver operating characteristic (ROC) and two‐graph ROC (TG‐ROC) analyses were performed on the inflammatory mediators and biomarkers that exhibited the highest area under the curve (AUC) values.

**Result:**

The fold change results demonstrated significant increases in CCL11, CCL5, IFN‐γ, and PDGF in the MCI/HC profile, along with a decrease in CCL4. The DDA/HC profile showed increases in Aβ40, *p* ‐Tau, t‐Tau, the *p* ‐Tau/Aβ42 ratio, CCL11, and PDGF, while decreases were observed in Aβ42, the Aβ42/Aβ40 ratio, CXCL8, and IL‐1Ra. In the ADD/MCI profile, *p* ‐Tau, the *p* ‐Tau/Aβ42 ratio, CCL3, and G‐CSF exhibited increased fold changes, whereas Aβ42, the Aβ42/Aβ40 ratio, CCL2, and the IFN‐γ ratio were decreased. CCL11 was able to discriminate between MCI and HC groups, while *p* ‐Tau effectively distinguished ADD from both MCI and HC.

**Conclusion:**

Our findings provide new insights into the role of neuroinflammation in AD, contributing to a deeper understanding of the dynamics of biomarkers and immune mediators in the pathophysiology of the disease.